# Reelin expression in brain endothelial cells: an electron microscopy study

**DOI:** 10.1186/s12868-015-0156-4

**Published:** 2015-03-24

**Authors:** Emma Perez-Costas, Erin Y Fenton, Hector J Caruncho

**Affiliations:** Department of Psychology, University of Alabama at Birmingham, College of Arts and Sciences, Campbell Hall 415, 1720 2nd Avenue South, Birmingham, Alabama 35294 USA; Division of Pharmacy, College of Pharmacy and Nutrition, University of Saskatchewan, 1B23 Health Sciences Building, 107 Wiggins Road, Saskatoon, SK S7N 5E5 Canada

**Keywords:** Blood-brain-barrier, Transcytosis, Brain capillaries

## Abstract

**Background:**

Reelin expression and function have been extensively studied in the brain, although its expression has been also reported in other tissues including blood. This raises the possibility that reelin might be able to cross the blood-brain barrier, which could be functionally relevant. Up-to-date no studies have been conducted to assess if reelin is present in the blood-brain barrier, which is mainly constituted by tightly packed endothelial cells. In this report we assessed the expression of reelin in brain capillaries using immunocytochemistry and electron microscopy.

**Results:**

At the light microscope, reelin immunolabeling appeared in specific endothelial cells in brain areas that presented abundant diffuse labeling for this protein (e.g., layer I of the cortex, or the *stratum lacunosum moleculare* of the hippocampus), while it was mostly absent from capillaries in other brain areas (e.g., deeper cortical layers, or the CA1 layer of the hippocampus). As expected, at the electron microscope reelin labeling was observed in neurons of the cortex, where most of the labeling was associated with the rough endoplasmic reticulum. Importantly, reelin was also observed in some endothelial cells located in small capillaries, which confirmed the findings obtained at the light microscope. In these cells, reelin labeling was located primarily in caveolae (i.e., vesicles of transcytosis), and associated with the plasma membrane of the luminal side of endothelial cells. In addition, some scarce labeling was observed in the nuclear membrane.

**Conclusions:**

The presence of reelin immunolabeling in brain endothelial cells, and particularly in caveolar vesicles within these cells, suggests that reelin and/or reelin peptides may be able to cross the blood-brain barrier, which could have important physiological, pathological, and therapeutic implications.

## Background

Reelin is a large extracellular matrix glycoprotein, which was originally discovered in the mouse brain [1]. This protein is secreted by Cajal-Retzius cells during brain development, regulating neural migration and dendritic spine maturation (see as reviews [2-5]). In the adult brain, reelin is expressed primarily in cortical and hippocampal GABAergic neurons as well as in cerebellar granule cells [6-10]. In the mature brain, it is involved in a variety of functions, including the ccontrol of neurotransmitter release [11,12], maturation and stabilization of dendritic spines [13-15], specification of the molecular identity of the distal dendritic compartment of pyramidal neurons [16], regulation of glutamate receptor homeostasis [17], and in the regulation of different aspects of adult hippocampal neurogenesis [18-25]. However, reelin expression is not restricted to the central nervous system (CNS), and has also been observed in the enteric nervous system [26], blood serum, liver, pituitary pars intermedia, and adrenal chromaffin cells [27], as well as in platelets [28], and lymphatic vessels [29,30]. The functional roles of reelin outside the CNS have been less studied, although it has been suggested that reelin enhances spreading of platelets on fibrinogen [28], and participates in the regulation of lymphatic vessel formation [30].

Some ultrastructural studies have focused primarily on the subcellular expression of reelin in neurons and neuropil within the CNS, where immunolabeling has been reported in the rough endoplasmic reticulum, axons, dendritic spines, and in the extracellular matrix [31-35]. However, as far as we know there are no reports on the possible expression of reelin in the main component of the blood-brain barrier, the brain endothelial cells. The expression of reelin in this particular type of endothelial cells, and in specific subcellular compartments within them, could be related to possible roles for this protein in the regulation of brain microvasculature (i.e., as it has been shown for lymphatic vessels), and/or mediating an interconnection between brain and peripheral tissues (i.e., being transported by transcytosis). In order to assess these possibilities we undertook an electron microscopy study of reelin immunolabeling in brain endothelial cells.

## Results and discussion

**Figure 1:**
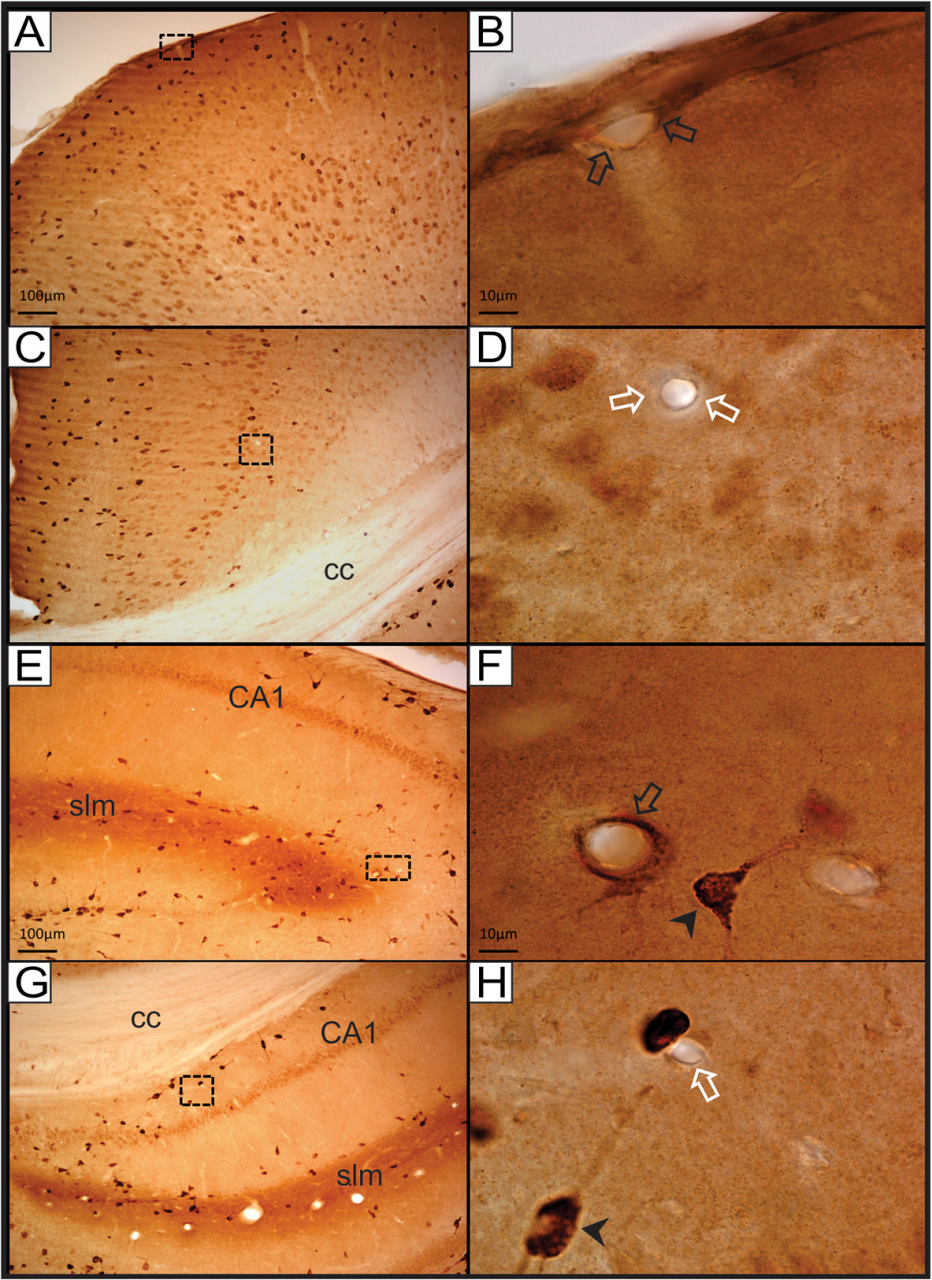
**Reelin labeling at the light microscope. A)** Reelin immunolabeling in superficial and mid-layers of the cortex. As expected, reelin-labeled neurons are present across different layers of the cortex, and diffuse labeling is mostly present in the superficial layers. **B)** High magnification of the area indicated with a dashed-line box in A. This image shows a reelin-immunolabeled blood capillary in layer I of the cortex (empty black arrows). **C)** Reelin labeling in deeper layers of the cortex. Scattered reelin-labeled neurons in these deeper layers present moderate labeling compared to superficial layers. **D)** High magnification image of the area indicated with a box in C. The capillary is negative for reelin labeling (empty white arrows). **E)** Reelin labeling in the lateral part of the rostral hippocampus. Note the row of lightly reelin-labeled neurons in the CA1, as well as the diffuse reelin immunolabeling in the *stratum lacunosum moleculare* (slm). **F)** High magnification image of an area neighboring the slm, corresponding to the dashed-line area indicated in E. Note the intense reelin labeling surrounding this capillary (empty black arrow). In addition there is a strongly labeled neuron in close proximity (black arrowhead). **G)** Reelin labeling in the medial part of the rostral hippocampus. The corpus callosum (cc) appears dorsally bordering the hippocampus. **H)** High magnification image of the dashed-line box area in G. Note the unlabeled capillary (empty white arrow) located in close proximity to a strongly labeled neuron (black arrowhead). **cc**: corpus callosum; **CA1**: Cornu Ammonis layer I; **slm**: stratum lacunosum moleculare. Scale bars: 10 microns in **A, C, G, E**; 100 microns in **B, D, F, H**.

In this study we focused on reelin immunolabeling in the adult rat cortex and hippocampus. As shown in previous studies [6-9,13,34,35], at the light microscope the most intense reelin labeling was observed in layer I of the cortex and in the *stratum lacunosum moleculare* of the hippocampus, where neuronal and diffuse labeling were observed (Figure 1). Interestingly, at higher magnification reelin labeling also appeared in some (but not all) capillaries within areas containing strong diffuse immunolabeling, such as layer I of the cortex (Figures 1A-B) and the *stratum lacunosum moleculare* of the hippocampus (Figures 1E-F). On the other hand, reelin-labeled capillaries were not observed in areas lacking diffuse immunostaining, such as deeper layers of the cortex, and the CA1 area of the hippocampus (Figures 1C-D, G-H). The fact that reelin immunostaining was only observed in some (but not all) capillaries within areas presenting strong diffuse labeling argues in favor of its specificity. In addition, this also suggests that a possible secretion and/or transport of reelin by endothelial cells may take place primarily in areas of heavy diffuse reelin staining (i.e., brain areas where reelin tends to accumulate in the extracellular matrix) [see reference 32].

**Figure 2:**
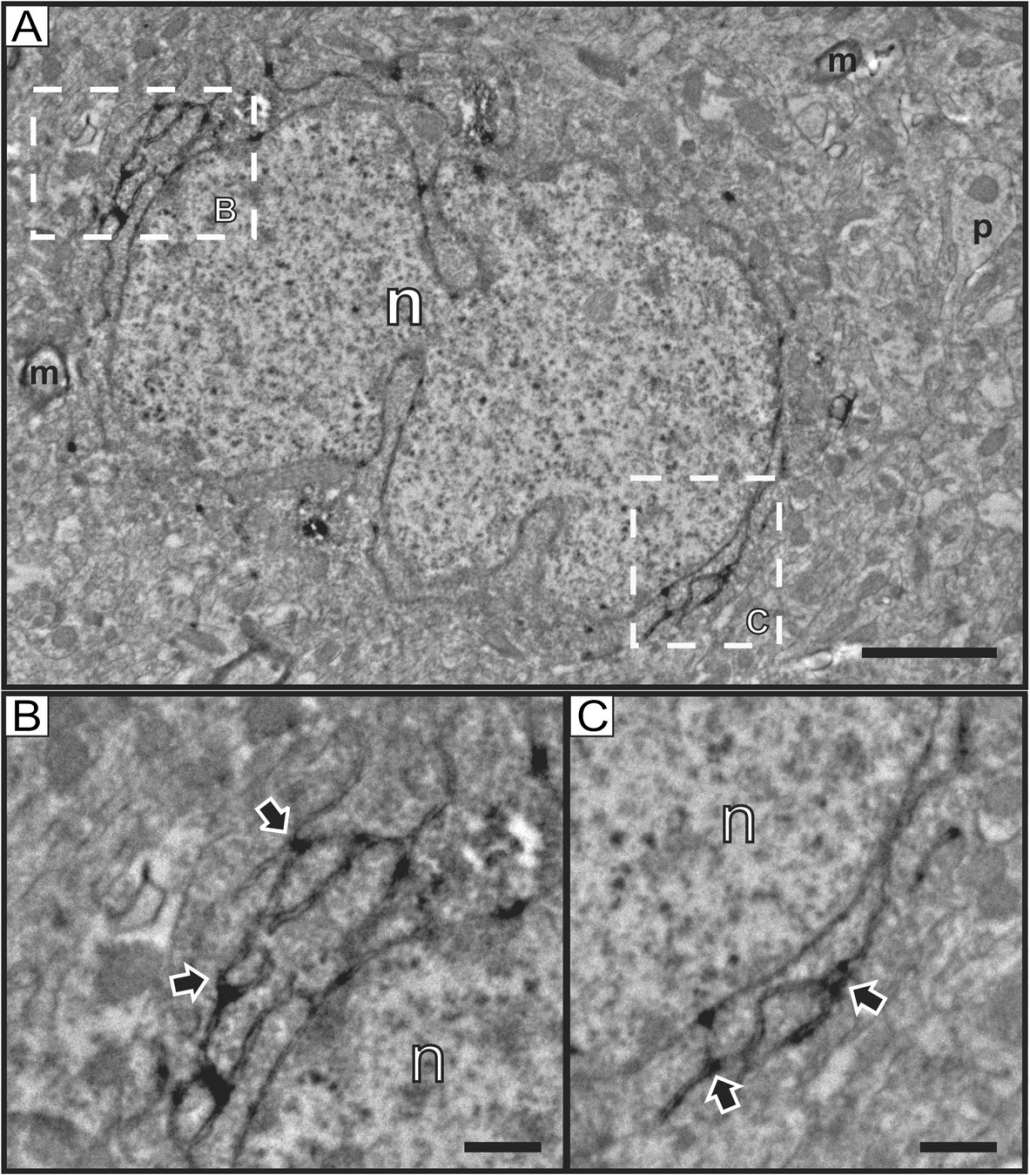
**Reelin neuronal labeling at the electron microscope. A)** Low magnification electron microscopy image of a reelin immunolabeled neuron in the cortex. **B-C)** Detail images of the areas indicated with dashed-line boxes in A. Note that reelin labeling is specifically located in the rough endoplasmic reticulum (outlined black arrows). **m**: myelinated process; **n**: nucleus; **p**: neuronal process. Scale bars: 2 microns in A; 0.5 microns in **B-C**.

**Figure 3:**
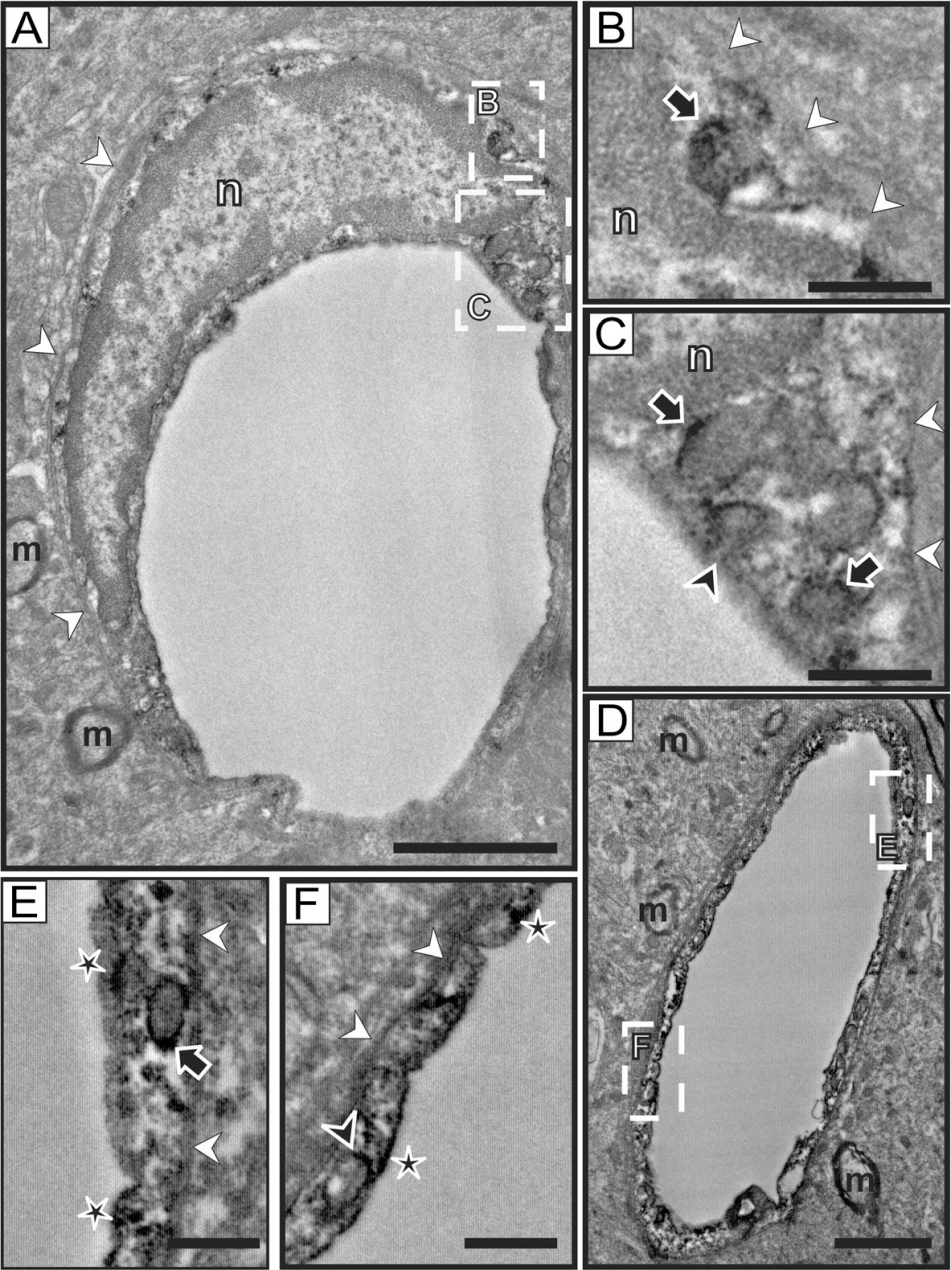
**Reelin labeling in endothelial cells. A)** Low magnification electron microscopy image of a reelin-labeled endothelial cell in the cortex. This endothelial cell presents a thin cytoplasm, which is clearly delimited by the basement membrane (white arrowheads) in the abluminal side. The nucleus (n) occupies most of the cell body of the endothelial cell. **B-C)** Details of the areas marked with dashed-lined boxes in A. In B, a reelin-labeled transcytosis vesicle (outlined black arrow) is located in a narrow cytoplasm area between the nucleus (n) and the cell membrane (white arrowheads). In C, a small cluster of reelin-labeled caveolar vesicles is shown (outlined black arrows), with one of them presenting the open neck towards the capillary lumen (outlined black arrowhead). Note also that the abluminal side (white arrowheads) is devoid of labeling. **D)** Reelin labeling in a small brain capillary in the cortex. This capillary is formed by the almost-continuous thin cytoplasm of a reelin-labeled endothelial cell. Note also the lack of labeling in myelinated processes (m) located in the vicinity of this capillary. **E-F)** Detail images of the areas indicated by dashed-line boxes in D. Reelin-labeled caveolar vesicles are present in different stages. A labeled caveolar vesicle in E (outlined black arrow) presents a well-delineated oval shape, while a labeled caveolar vesicle in F that is located in close proximity to the luminal side of the capillary is about to form the open neck (outlined black arrowhead). Note also the presence of reelin labeling in the luminal side of the membrane (outlined black stars), and the lack of labeling in the abluminal side (white arrowheads). **m**: myelinated process; **n**: nucleus. Scale bars: 2 microns in **A**, **D**; 0.5 microns in **B-C** and **E-F**.

**Figure 4:**
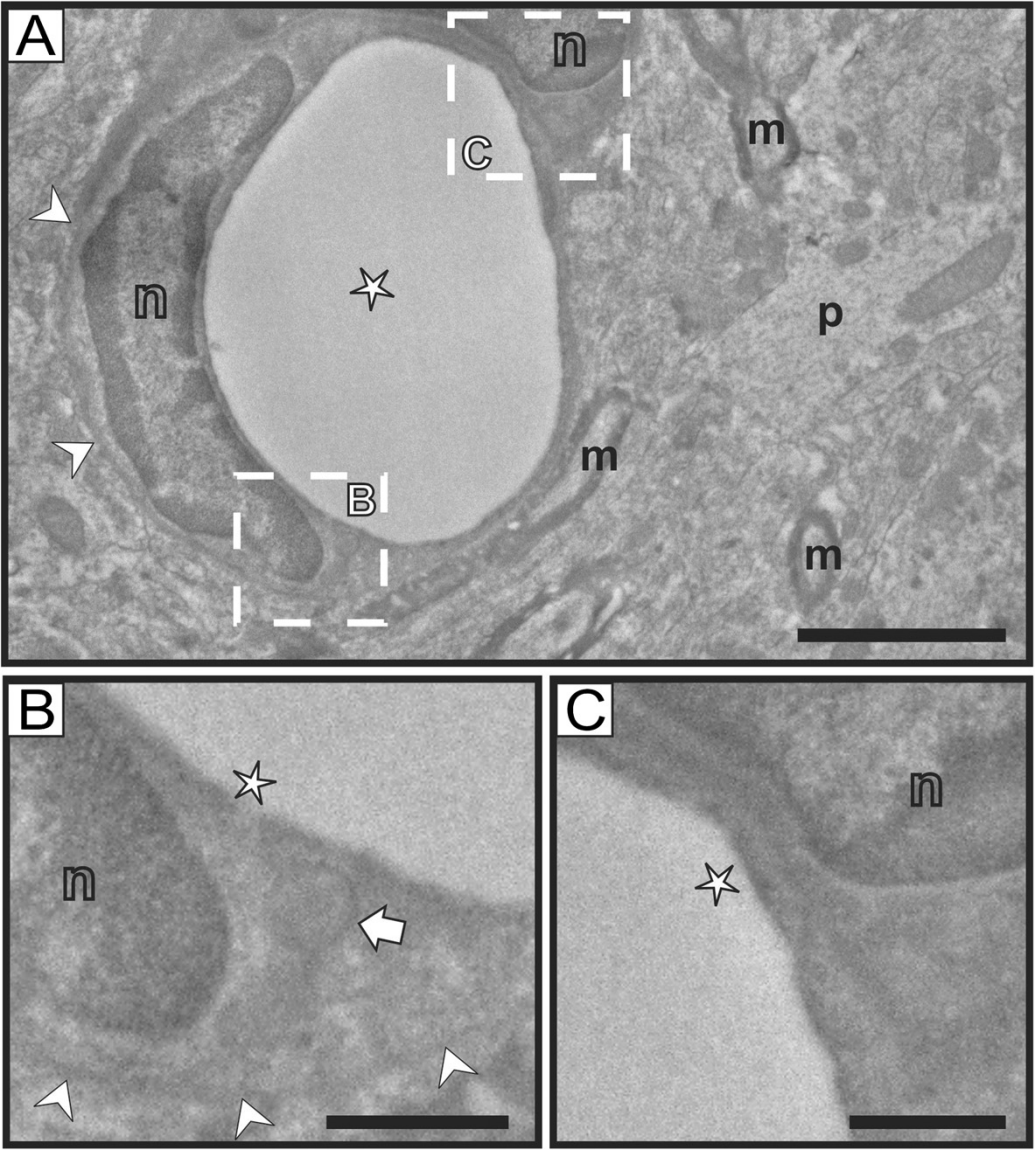
**Reelin labeling is not present in all endothelial cells. A)** Low magnification image of an unlabeled brain capillary in the cortex. The wall of this brain capillary is lined by at least two endothelial cells, as shown by the presence of their nuclei (n). Note also the presence of unlabeled myelinated (m) and non-myelinated (p) neuronal processes. The basement membrane was also clearly present (white arrowheads). **B-C)** Detail images of areas indicated with dashed-lined boxes in A. In B, a caveolar vesicle is clearly unlabeled (white arrow). In B and C, the luminal side of the endothelial cell membrane is also devoid of labeling (white stars). **m**: myelinated process; **n**: nucleus; **p**: neuronal process. **White arrowheads** indicate the membrane in the abluminal side Scale bars: 2 microns in A; 0.5 microns in **B-C**.

Our electron microscopy study confirmed and extended our findings. As expected, reelin immunostaining was found in neurons, where labeling was located in discrete regions of the rough endoplasmic reticulum (Figures 2A-C), which is in agreement with previous studies [31-35], and is also consistent with the fact that reelin is an extracellular matrix protein expressed through the secretory pathway. Importantly, we have also confirmed that some endothelial cells associated with small capillaries contain reelin immunostaining (Figure 3), while others appear devoid of labeling (Figure 4). High magnification electron micrographs allowed us the identification of the subcellular distribution of reelin labeling, which was mostly located inside vesicles of transcytosis (Figures 3B-C, E-F). In fact, we were able to observe almost all stages of the transcytosis vesicles, from the formation of one of these vesicles accumulating reelin immunostaining (Figure 3B), to reelin-labeled caveolar vesicles located close to the lumen of the capillary or even showing the opening neck (Figures 3C, E-F). In addition, reelin labeling was also found in the endothelial plasma membrane bordering the capillary lumen (Figures 3E-F), but not in the abluminal side (Figures 3B-C, E-F). Furthermore, we found some punctual labeling in the nuclear envelope, which may suggest a possible synthesis of reelin by endothelial cells; although we did not observe immunolabeling in the Golgi complex of these cells, which is poorly developed. Finally, we also found capillaries completely devoid of reelin labeling (Figure 4) in the same electron microscopy sample of the cortex, confirming our light microscopy observation that reelin appears in some (but not all) capillaries within the same area.

Although out of the scope of the present work, the presence of reelin immunolabeling in brain endothelial cells suggests that this protein may play a role in regulating the formation of brain microvasculature through a mechanism similar to the previously reported for lymphatic vessels [30]. Interestingly, it has been previously reported that in the cerebral cortex there are differences in the orientation of the cerebral vasculature between reeler and wild type mice, which suggests that reelin may be involved in blood vessel development [36]. However, additional experiments will be necessary to test whether reelin plays a role in the formation of brain microvasculature.

The fact that reelin labeling was observed in caveolar vesicles in different stages (i.e., from vesicles just formed close to the abluminal side of the plasma membrane, to others developing an open neck to the capillary lumen) indicates a possible process of transcytosis, which suggests that reelin may cross the blood-brain barrier. Alternatively, reelin transport might be also from the brain parenchima to the periphery, or may occur in both directions. Interestingly, it has been shown that apolipoprotein-E receptor-2 (ApoER2), which together with the very-low-density-lipoprotein receptor (VLDLR) constitutes the receptor for reelin, tends to co-immunoprecipitate with caveolin 1, and to localize in caveolae [37,38]. Taking together our findings and these previous reports, it is possible that the crossing of the blood-brain barrier might be accomplished via a receptor-mediated mechanism that includes ApoER2. In addition, it has been shown that the adaptor protein DAB1 also tends to cluster within specific membrane domains (i.e., in detergent-resistant membrane fractions such as lipid rafts and caveolar vesicles) [39,40], that reelin induces the clustering of its own receptor Apolipoprotein ER2 and other proteins [41,42], and that the sorting of these proteins to different membrane domains yields differential endocytosis of reelin [43]. We have recently shown that deficits in reelin expression result in changes in membrane protein clustering in peripheral lymphocytes [44], and that the addition of recombinant reelin to synaptosomes increase protein expression and alters membrane protein clustering [45,46]. Interestingly, there is a decrease in reelin expression in the brain and periphery of patients diagnosed with depression [47,48], and we have found alterations in membrane protein clustering in lymphocytes from patients with depression, which correlated with therapeutic response to antidepressants [49,50]. All these evidence point towards an important role for reelin in membrane protein clustering in both brain and periphery. The presence of reelin in caveolar vesicles of brain endothelial cells suggests a possible role for this protein in the regulation of the crosstalk between brain and immune system, and in the perturbation of this crosstalk in several psychiatric disorders. Although the specific consequences of reelin downregulation in psychiatric disorders are still unknown, and it should be also taken into account that reelin deficits could be associated with brain cell malfunction independent of its association with the blood-brain barrier.

It does not escape to us that if reelin was able to cross the blood-brain barrier, it would be easier to design reelin peptides as possible therapeutic agents for psychiatric disorders. This is of particular interest taking into account that overexpression of reelin has been shown to prevent behavioral phenotypes in animal models of mood and psychotic disorders [51].

## Conclusions

Reelin immunolabeling is specifically present in caveolar vesicles within endothelial cells located in brain areas that present strong reelin labeling in the extracellular matrix. In addition, electron microscopy images evidence that reelin-labeled caveolar vesicles can be observed from their formation to the opening of their membrane towards the capillary lumen. Altogether, these observations suggest that reelin and/or reelin peptides might cross the blood-brain barrier, which could have important physiological, pathological, and therapeutic implications.

## Methods

### Tissue preparation

Rat brain samples were obtained from adult male rats (Charles River, Montreal, Canada) weighting between 225 and 250 grams at arrival to the animal facility. Animals were kept in a 12/12 hour night/day cycle with water and food ad libitum until endpoint. The rat brain tissue used in this study belonged to a stock of spare samples prepared for light and electron microscopy. Procedures for tissue collection were approved by the University of Saskatchewan Committee on Animal Care and Supply, in strict accordance with the Canadian Council on Animal Care Guidelines. The protocols used in this study were also in accordance with the Institutional Animal Care and Use Committee (IACUC) guidelines at the University of Alabama at Birmingham, and the National Institutes of Health guidelines for the care and use of animals in experimental procedures (USA). Rats were deeply anesthetized with sodium pentobarbital and transcardially perfused with saline solution (0.9% NaCl), followed by 4% paraformaldehyde in 0.1 M phosphate buffer (PB), pH 7.4. Brains were post-fixed in 4% paraformaldehyde for 72 hr at 4°C, thoroughly rinsed in PB and stored in PB containing 0.02% sodium azide. Brain sections (50 micron thick) were obtained in the coronal plane on a vibrating microtome. Sections at the level of the rostral hippocampus were used for light and electron microscopy.

### Light Microscopy immunohistochemistry

Free-floating sections were thoroughly rinsed in 0.01 M phosphate buffered saline (PBS) pH 7.4, and subsequently treated with 0.3% (v/v) H_2_O_2_ in PBS for 30 min to block endogenous peroxidase activity. They were then pre-incubated for 30 min in a blocking solution containing 0.3% Triton X-100, 5% normal horse serum (NHS; Vector Laboratories, Burlingame, CA, USA), and 1% bovine serum albumin (BSA) dissolved in PBS in order to block any non-specific antibody binding. The sections were then incubated with a mouse monoclonal anti-reelin antibody (Millipore clone G-10 Cat# MAB5364, RRID:AB_2179313; diluted 1:2000, 48 hr, at 4°C). This incubation was followed by three rinses in PBS and incubation for 1 hour at room temperature with a biotinylated horse anti-mouse secondary antibody (Vector Laboratories) diluted 1:200 in PBS containing 0.3% (v/v) Triton X-100. After several rinses in PBS, the sections were incubated with a peroxidase-coupled avidin-biotin complex (Vecta-Stain Elite ABC Kit, Vector Laboratories) for 90 minutes at room temperature. To visualize the immunolabeling, the sections were developed using a solution of 0.033% 3,3’-diaminobenzidine (DAB) and 0.00786% H_2_O_2_ prepared in PBS. The reaction was stopped using PBS as a rinse. The sections were then mounted onto slides, allowed to dry overnight, dehydrated in ethanol, cleared in xylene and coverslipped using Entellan resin (Millipore, USA). Omission of the primary antibody resulted in a complete lack of immunostaining (not shown). In addition, labeling with a different anti-reelin primary antibody (*Reln AB142*) yielded similar results than those obtaining using the G-10 anti-reelin antibody (data not shown).

Light microscopy images of reelin immunolabeling were obtained using a computerized Nikon E800 microscope.

### Electron Microscopy immunohistochemistry

Free-floating sections were treated with 1% sodium borohydride in PBS for 15 minutes and thoroughly rinsed in PBS (5 rinses, 5 min each). Endogenous peroxidase was blocked by immersing the sections in a solution of 5% hydrogen peroxide in PBS for 30 minutes, which was followed by 5 rinses (5 min each) in PBS. Afterwards, to block non-specific binding sites in the sections, a blocking solution of 10% NHS prepared in PBS containing 0.1% Triton X-100 was applied for one hour. The sections were then incubated for 72 hours at 4°C with a mouse monoclonal anti-reelin antibody (Millipore clone G-10 Cat# MAB5364, RRID:AB_2179313clone) diluted 1:1000 in PBS containing 5% NHS. After 5 rinses (5 min each) in PBS, the sections were incubated with a biotinylated horse anti-mouse secondary antibody (Vector Laboratories) diluted 1:400 for 45 minutes at room temperature, and rinsed in PBS (5 times, 5 min each). Finally, the sections were incubated for 45 minutes with a peroxidase-coupled avidin-biotin complex (Vecta-Stain Elite ABC Kit, Vector Laboratories), rinsed in PBS, and the reaction was developed using a 3-3’ diaminobenzidine-peroxidase kit (DAB-peroxidase kit, Vector Laboratories). The reaction was stopped by rinsing the sections in PBS, and the sections were further rinsed in PB prior to embedding for electron microscopy. After immunocytochemistry, all sections for electron microscopy were postfixed overnight at 4°C in 2% glutaraldehyde prepared in PB.

Sections were embedded for electron microscopy as previously described in Perez-Costas et al [52], with some modifications. After several rinses in PB, immunolabeled sections were immersed in a solution of 1% osmium tetroxide in PB for 1 hour at room temperature, rinsed in PB two times (5 min each), and gradually dehydrated in a series of ethanol (30%, 50%, 70% v/v of ethanol in milli-Q ultrapure water). After this partial dehydration, sections were stained with a solution of 1% uranyl acetate in 70% ethanol for 1 hour at room temperature, and then dehydration was completed by immersing the sections gradually in 90% and 100% ethanol (3 baths, 5 min each). After dehydration the sections were cleared in propylene oxide (2 baths, 5 min each), progressively infiltrated at room temperature with Epon resin diluted in propylene oxide (1:1 v/v propylene oxide/resin, 30 minutes; followed by v/v 1:2 propylene oxide/resin, 1 hour). Finally, the sections were immersed in pure Epon resin overnight at 4°C, transferred to freshly-prepared Epon resin for flat-embedding, and allowed to polymerize in an oven set at 60°C for 72 hours. In order to select the regions of interest (i.e. frontal cortex), the embedded sections were visualized on a Nikon Eclipse 50 I light microscope, carefully identifying the cortical region of interest, and re-dissecting this region for ultramicrotomy. Ultrathin sections (90 nm thick) were obtained using a Leica UC6 ultramicrotome (Leica Microsystems, Wetzlar, Germany) and mounted on copper-slot grids (Electron Microscopy Sciences, USA). Immunolabeled cortex samples were then observed and photographed using a transmission electron microscope (H-7650, Hitachi, Japan) equipped with a Hamamatsu Orca HR digital camera (Hamamatsu, Japan).

### Photomontage and lettering

Adjustment of brightness/contrast and figure plate lettering were done using Corel Draw X5 (Corel Corporation, Ottawa, Canada)
